# Avoiding the Formation of Bubbles and Pits in Buffered Chemical Polishing for the Niobium Superconducting Cavity Through Adjusting the Acid Ratio

**DOI:** 10.3390/ma18050960

**Published:** 2025-02-21

**Authors:** Zheng Wang, Jinfang Chen, Yue Zong, Shuai Xing, Jiani Wu, Yawei Huang, Xiaowei Wu, Zhejia Xu, Xuhao He, Xiaohu Wang, Xuan Huang, Zhaoxi Chen, Xuerong Liu, Dong Wang

**Affiliations:** 1 Shanghai Institute of Applied Physics, Chinese Academy of Sciences, Shanghai 201800, China; wangzheng@sinap.ac.cn (Z.W.); huangxuan@sinap.ac.cn (X.H.); 2 University of Chinese Academy of Sciences, Beijing 101408, China; 3 Shanghai Advanced Research Institute, Chinese Academy of Sciences, Shanghai 201204, China; zongy@sari.ac.cn (Y.Z.); xings@sari.ac.cn (S.X.); wujn@sari.ac.cn (J.W.); wangd@sari.ac.cn (D.W.); 4 Center for Transformative Science, ShanghaiTech University, Shanghai 201210, China; huangyw2@shanghaitech.edu.cn (Y.H.); xuzhj2022@shanghaitech.edu.cn (Z.X.); hexh2022@shanghaitech.edu.cn (X.H.); wangxh4@shanghaitech.edu.cn (X.W.); chenzhx@shanghaitech.edu.cn (Z.C.); liuxr@shanghaitech.edu.cn (X.L.); 5 Zhangjiang Laboratory, Shanghai 201210, China; wuxw@zjlab.ac.cn

**Keywords:** superconducting radio-frequency cavity, buffered chemical polishing, acid ratio, pit, bubble, viscosity

## Abstract

Buffered chemical polishing (BCP) is an important and widely used polishing technique for superconducting radio-frequency (SRF) cavities made of niobium. A common problem encountered during BCP is the formation of bubbles and W-shaped pits on the cavity surface, which may severely limit the RF performance. We report a method to address the problem of W-shaped pits through optimizing the BCP acid ratio. We systematically investigate the effect of the BCP acid ratio through sample and cavity BCP experiments and determine an optimal ratio for the three acids. The new BCP recipe with the optimal acid ratio is verified through the development of niobium cavities with several different shapes, which are shown to be free of pits and demonstrate excellent RF performance; notably, several 3.9 GHz nine-cell cavities present unprecedented accelerating gradients. Furthermore, the findings suggest a simple pit-free BCP recipe that does not require $H_{3}{\mathrm{PO}}_{4}$, using only HF and ${\mathrm{HNO}}_{3}$. The method proposed in this study is also appropriate for suppressing pit formation with other acid mixtures or when polishing other metal objects.

## 1. Introduction

Superconducting radio-frequency (SRF) cavities are key devices for superconducting accelerators, which are now widely employed in accelerator-based light sources, neutron sources, and particle colliders [1,2]. Buffered chemical polishing (BCP) is a simple, widely used, and reproducible chemical polishing method for SRF cavities to provide high RF performance, which involves removing damaged or contaminated surface layers. BCP technology has been applied in the European XFEL project for the final polishing of 400 1.3 GHz cavities [3] and for both bulk and final polishing of all 3.9 GHz cavities [4], in the European Spallation Source project for polishing all medium beta 704.42 MHz superconducting cavities [5], as well as in the HERA for polishing 500 MHz cavities [6]. For the Shanghai hard X-ray FEL facility (SHINE) [7], apart from several hundred TESLA-type [8] high-Q cavities, BCP is required for processing the 1.3 GHz nine-cell cavity with twin fundamental power couplers for the injector cryomodule, as well as the 16 3.9 GHz nine-cell cavities for the two third-harmonic cryomodules [9,10].

The classic BCP acid mixture consists of hydrofluoric acid (HF, 49%), nitric acid (${\mathrm{HNO}}_{3}$, 69%), and phosphoric acid ($H_{3}{\mathrm{PO}}_{4}$, 85%) mixed in the ratio 1:1:2 or 1:1:1 by volume [11,12,13,14,15]. The nitric acid in the acid mixture reacts with niobium to form niobium oxide and produces nitric oxide (NO) gas, the hydrofluoric acid dissolves the niobium oxide to polish the superconducting cavity, and the phosphoric acid acts to slow down or control the reaction rate and inhibit turbulence. As BCP is an exothermic etching process, the acid needs to be stirred and cooled during polishing. Usually, the acid temperature is controlled below 15 °C to prevent excessive hydrogen absorption by niobium [16,17].

The uniformity of removal and the final surface smoothness achieved through BCP are very important for superconducting cavities [18,19,20,21,22]. To achieve a more uniform removal and a smoother final surface of Nb cavities, structural optimizations of BCP devices have been investigated by several laboratories worldwide, such as designing a flow diverter [23] or adding a rotating setup [24,25], among other considerations. Furthermore, BCP process parameters have been studied, such as using a lower acid temperature [18] or a proper acid flow rate [24]. In particular, methods such as agitation, rotation, or forced convection have been used to avoid patterns such as grooves, stripes, or bubble traces [13,19,26].

However, we found that many bubbles gradually formed and attached to the cavity surface when the cavity was polished with an acid mixture at the ratio 1:1:2, and the higher etching rate at the bubble attachment locations led to the formation of W-shaped pits [27]. Two main directions have been proposed to prevent the formation of W-shaped pits on the cavity surface. One is to remove bubbles through stirring or vibration, while the other involves reducing the bubble production rate while decreasing the viscosity of the acid, for example, by changing the acid ratio and/or lowering the acid temperature.

In this study, we systematically investigate the effects of the ratio of the three acids on bubbles and W-shaped pits through sample BCP experiments and cavity BCP verification. First, we describe the experimental method and our apparatus built for the BCP acid ratio experiments. Then, we choose and verify a superior BCP acid ratio. Finally, we fully optimize the BCP polishing process through single-cell cavities and apply it to the BCP treatment of SHINE nine-cell cavities.

## 2. Experiments and Results

### 2.1. Experimental Methods

#### 2.1.1. Sample BCP Experiments

A set of clean niobium samples (8 mm × 5 mm × 3 mm in dimension, RRR ≥ 300) was prepared, including smooth large-grain (NLxx-marked), smooth fine-grain (Fxx-marked), and rough fine-grain (NFxx-marked) samples. The sample preparation details can be found in the literature [27]. The slight roughness differences between three types of samples originated from the original niobium sheets. Most of the NLxx samples are single-crystal samples. The samples were numbered with an electro-engraving pen, weighed with an electronic balance, measured for thickness with a micrometer, and optically inspected using a high-definition camera in an ISO7-class clean room prior to BCP, in order to record the surface topography of the samples before polishing.

**Figure 1:**
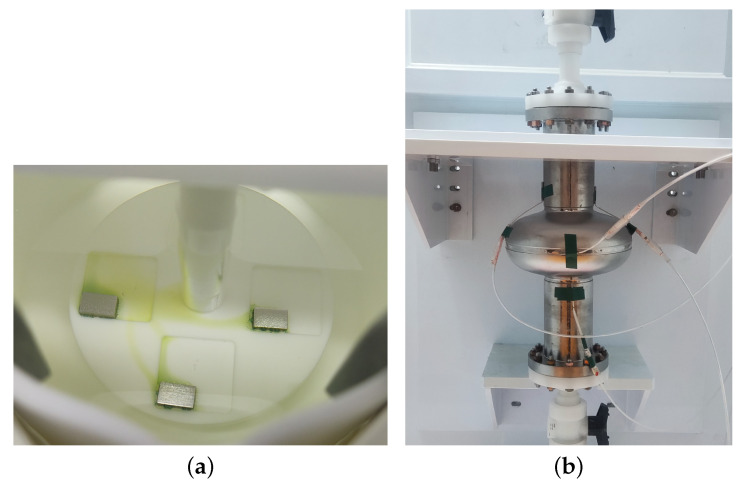
(**a**) Sample BCP setup and (**b**) cavity BCP-I device at Wuxi platform.

We prepared the BCP mixed-acid solution (400 mL) and injected it into the beaker of the sample BCP setup. The goal acid temperature, rotor speed (i.e., acid flow velocity), and other parameters were set first. Then, a set of samples were put into the setup and polished, including one each of slightly rough fine-grain NFxx, large-grain NLxx, and fine-grain Fxx, as shown in Figure 1a. The whole polishing process was observed and recorded using a camera. After polishing, the samples were rinsed with ultra-pure water until they reached neutral pH. Finally, the samples were rinsed again with ultra-pure water in an ISO7 clean room and blown dry with nitrogen.

The samples were weighed, their thickness was measured, and they were optically inspected again to record the surface topography of the samples after polishing. The average polishing rate of the samples was calculated from the mass reduction before and after BCP. We compared the surface morphology of the samples before and after polishing, together with the observations made during the polishing process, in order to determine the status of pit formation on the niobium surface under the corresponding polishing conditions.

#### 2.1.2. Cavity BCP Experiments

At the Wuxi platform, BCP processing is available for both the inner and outer surface (BCP-O) of a cavity [27]. The cavities were weighed with an electronic scale, their thickness measured with an ultrasonic thickness meter, and their inner surface optically inspected with an optical inspection system developed by KEK, Tsukuba, Japan [28], in order to record the cavity surface state before and after polishing.

Figure 1b shows the apparatus used for cavity inner-surface BCP (BCP-I). The cavity is installed into the BCP-I device, and several thermal sensors are mounted at iris and equator regions to monitor the temperature during the BCP process. Parameters such as the acid temperature, flow rate, and polishing time can be set before polishing. After polishing, the cavity was subjected to HPR and dried in the clean room.

The average polishing rate of the cavity was calculated from the weight before and after BCP-I. The polishing quality can be determined by comparing the inner surface morphology of the cavity before and after polishing, for instance, whether pits are formed on the cavity surface.

### 2.2. Changing the Acid Ratio

In previous studies, we have proposed two solutions to the problem of W-shaped pit formation during BCP processing; among them, one is to remove bubbles via vibration, while the other involves increasing the dissolution rate of NO gas in the acid and reducing the viscosity of the acid, thereby reducing the bubble production rate [27]. The former requires installing a mechanical device to eliminate bubbles, whereas the latter can be realized by simply changing the acid ratio and polishing parameters.

#### 2.2.1. Effect of Ratio of Three Acids

A. Phosphoric Acid

Previous experimental studies have shown that, with a lower phosphoric acid ratio of N ≤ 1—where N is a positive number and HF (49%):${\mathrm{HNO}}_{3}$ (69%):$H_{3}{\mathrm{PO}}_{4}$ (85%) = 1:1:N—no bubbles and W-shaped pits appeared on the sample surface during BCP, while, when N > 1, bubbles started to form on the sample surface [27]. In the range 1 < N < 4, significant W-shaped pit formation was observed on the sample surfaces.

With a higher phosphoric acid ratio in the BCP acid mixture, the mixed acid will become more viscous [29,30], which will hinder gas diffusion or bubble movement [31,32,33,34], thus favoring the aggregation of gas and attachment of bubbles to the niobium surface. Furthermore, the disturbance and turbulence of acids on the niobium surface will be suppressed as well [15,35,36], which causes the bubbles to more stably attach to the surface. Therefore, a lower phosphoric acid ratio in the BCP acid mixture is beneficial for preventing bubble formation and attachment.

B. Nitric Acid

Concerning the nitric acid ratio, we take the ratio of 1:1:1 as a reference. The effect of the acid’s temperature on bubbles and W-shaped pits is significant, and the acid’s temperature on the cavity surface may vary over a wide range during BCP [27]. In order to confirm whether the new BCP acid ratio can polish the sample surface free of bubbles and W-shaped pits at both low and room temperatures, the experiments were divided into two groups: one with a low acid temperature (at 8 °C) and the other close to room temperature (at around 20 °C).

The polishing parameters of the sample BCP were set to be consistent with the phosphoric acid ratio experiments [27]; that is, acid temperature of 8 °C or 20 °C, rotor speed of 300 RPM (i.e., acid flow velocity on the sample surface at around 1.3 cm/s), and polishing time of 5 min × 2 (i.e., after the samples are polished for 5 min, they are turned upside down and polished for another 5 min), and the other conditions are fixed. These parameters for the subsequent sample BCP experiments are the same in this study, unless otherwise stated.

The experiments showed that, for the low-temperature experimental groups (at 8 °C), when the nitric acid ratio was sequentially 1:$\frac{1}{3}$:1 (i.e., 3:1:3), 1:$\frac{1}{2}$:1 (i.e., 2:1:2), 1:1:1, 1:2:1, 1:3:1, 1:4:1, 1:6:1, and 1:8:1, no bubbles or W-shaped pits were observed to form during polishing for all cases. Even when the acid temperature was increased from 8 °C to 20 °C, no bubbles or pits appeared on the sample surface.

**Figure 2:**
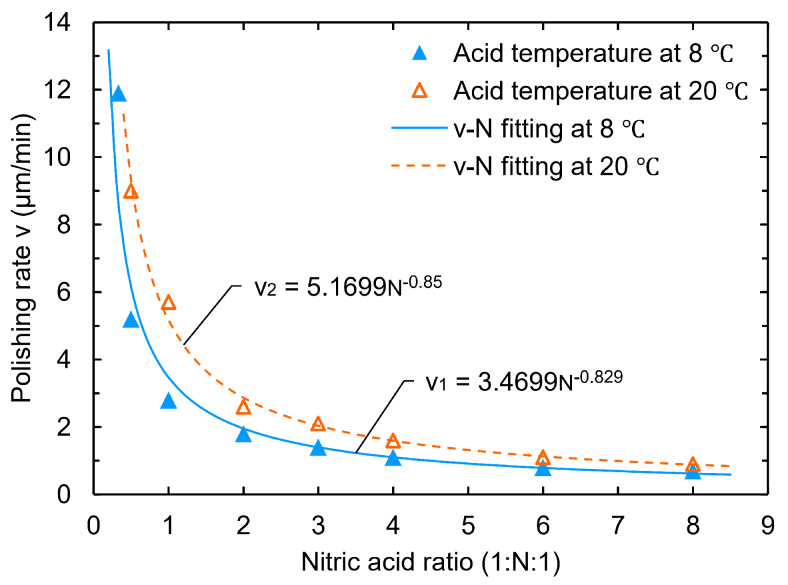
Effect of nitric acid ratio on polishing rate.

In addition, we found that the polishing rate decreased as a power function with the nitric acid ratio in the experiments, as shown in Figure 2. The estimated polishing rate error was less than 5%, which mainly arose from the sample size measurement. For this reason, we tried to remove the phosphoric acid to check the ability to reach the goal polishing rate for the cavity at around 1 μm/min. At a low temperature of 8 °C, the polishing rate was about 2.8 μm/min for the ratio 1:4:0 and 1.1 μm/min for the ratio 1:9:0, which is indeed a significant decrease in the reaction rate. Moreover, no bubbles or W-shaped pits were formed on the samples during the polishing process at acid temperatures of 8 °C, 20 °C, and 25 °C. These findings suggest a simple pit-free BCP recipe that does not require $H_{3}{\mathrm{PO}}_{4}$, using only HF and ${\mathrm{HNO}}_{3}$.

In the acids ratio (HF:${\mathrm{HNO}}_{3}$:$H_{3}{\mathrm{PO}}_{4}$) 1:N:1 series, the viscosities of the BCP mixed acids were all small, due to the low proportion of the highly viscous phosphoric acid [30,37,38,39]. In this case, gases and bubbles can easily diffuse [31,32,33,34], thereby making it difficult for bubbles to form and attach onto the niobium surface. For example, with the ratio 1:1:1, no bubble formation was observed on the sample surface during polishing; however, the acid exchange at the sample bottom-face was relatively slow, which hinders gas diffusion, prone to bubble formation. Then, bubbles may discharge from the bottom. Among them, some leave from the acids, while others merge to form large bubbles around the samples [27]. This also implies that a higher acid flow velocity is helpful to inhibit gas saturation and bubble formation on the niobium surface.

A high nitric acid ratio decreases the polishing rate and the NO gas production rate, while increasing the gas dissolution rate [40], thereby leading to less undissolved gas and bubble formation, which effectively inhibits the formation of bubbles and W-shaped pits. In our experiments, an acid ratio (HF:${\mathrm{HNO}}_{3}$:$H_{3}{\mathrm{PO}}_{4}$) of 1:N:1 led to sample surfaces which were free of bubbles and W-shaped pits.

C. Hydrofluoric Acid

**Figure 3:**
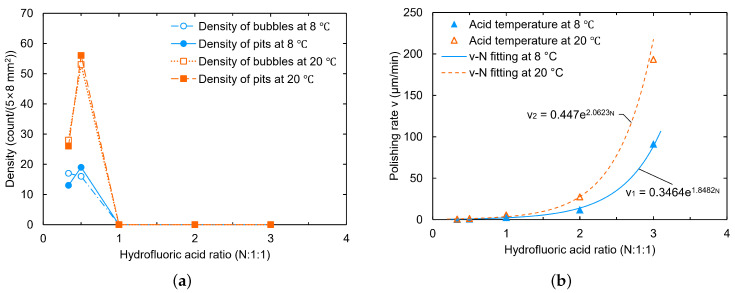
Effect of hydrofluoric acid ratio on bubble and pit density (**a**) and polishing rate (**b**).

Hydrofluoric acid plays the role of dissolving niobium oxide during the BCP process. In our experiments, we gradually increased the hydrofluoric acid ratio from $\frac{1}{3}$:1:1 (i.e., 1:3:3) to 3:1:1. As a result, the sample surfaces at different HF ratios gradually changed from presenting W-shaped pits to being free of pits, respectively. The polishing rate shows an exponential trend with the hydrofluoric acid ratio, as shown in Figure 3. The estimated density uncertainty of the bubbles and W-shaped pits should be less than 15%, which arises from the small number of bubbles and pits that are not visible.

**Figure 4:**
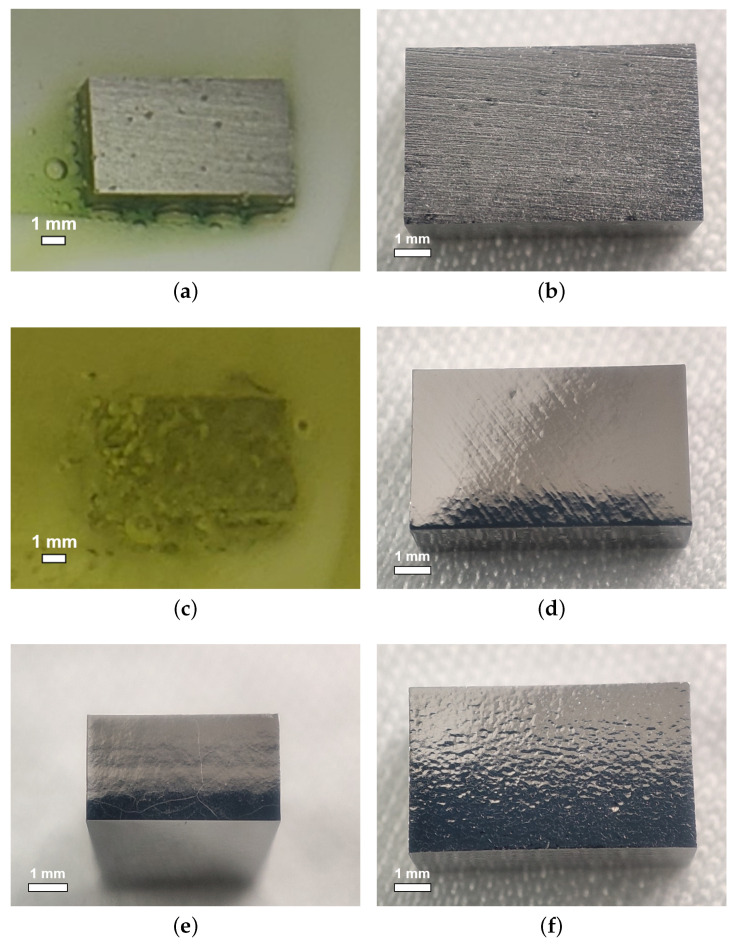
The surface states of large-grain sample NL161 during (**a**) and after (**b**) polishing at an acid ratio of $\frac{1}{2}$:1:1; large-grain sample NL160 during (**c**) and after (**d**) polishing at a ratio of 3:1:1; (**e**) grain boundaries on the side face of NL160; (**f**) significant scale-like pit structures on fine-grain sample NF160. The acid temperature for polishing was set at 8 °C.

As shown in Figure 4a,b, at the ratio of $\frac{1}{2}$:1:1, small bubbles attached to and W-shaped pits formed on the sample NL161. Meanwhile, at the ratio of 3:1:1, the niobium samples reacted violently with the acid, as shown in Figure 4c, in which the samples shook and presented a boiling phenomenon on the surface, producing a large number of bubbles and yellow-green products. No W-shaped pit or large grain-boundary step was observed on the surface of sample NL160 after polishing (see Figure 4d,e), and calculation revealed the polishing rate to be about 91.4 μm/min at 8 °C. However, a larger ratio of hydrofluoric acid also leads to the formation of scaly pit structures on the sample surface, especially for the fine-grained sample (see Figure 4f), which is unfavorable for acquiring a smooth surface.

A high HF ratio significantly increases the polishing rate and NO gas production rate, which promotes bubble formation. Furthermore, the low viscosity of the BCP mixed acid allows gas and bubbles to diffuse easier, thereby lowering bubble attachment to the surface. Furthermore, the rapid generation of gas or bubbles leads to strong disturbances on the sample surface [41], further promoting the rapid detachment of bubbles. Therefore, a high hydrofluoric acid ratio is favorable to avoid bubble attachment.

Nb samples polished at an acid ratio of 3:1:1 showed smaller grain-boundary steps than those polished at a ratio of 1:1:2 or 1:1:$\frac{12}{5}$ [27], which suggests a direction to reduce grain-boundary steps; that is, BCP with a high etching rate may suppress the etching difference between different grain orientations, in agreement with the literature [17]. However, the larger-reaction exotherm may lead the polishing process to become out of control.

In summary, at an HF ratio with N ≥ 1 (HF:${\mathrm{HNO}}_{3}$:$H_{3}{\mathrm{PO}}_{4}$ = N:1:1), no bubble or W-shaped pit was observed on the sample surface. A higher hydrofluoric acid ratio is favorable to avoid bubble attachment but may create pit structures and lead to difficulty in controlling the polishing process.

D. Other Acid Ratios

**Figure 5:**
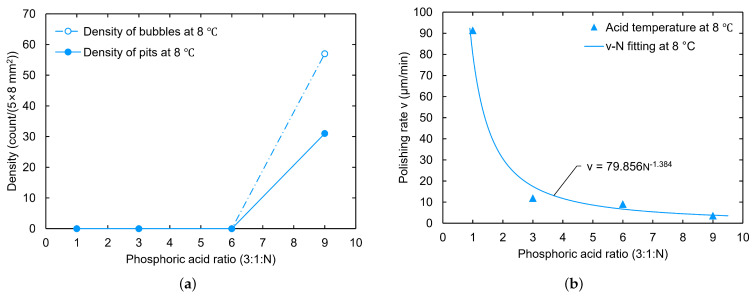
Effect of phosphoric acid ratio on bubble and pit density (**a**) and polishing rate (**b**) at an acid ratio (HF:${\mathrm{HNO}}_{3}$:$H_{3}{\mathrm{PO}}_{4}$) of 3:1:N.

As the acids ratio of 3:1:1 brings about a very fast reaction, we increased the phosphoric acid ratio to reduce the reaction rate, for which the experimental results are shown in Figure 5. When the phosphoric acid ratio was gradually increased from 3:1:1 to 3:1:9, bubbles and W-shaped pits appeared on the surface of the sample. This reconfirms that decreasing the phosphoric acid ratio prevents the formation and attachment of bubbles.

**Figure 6:**
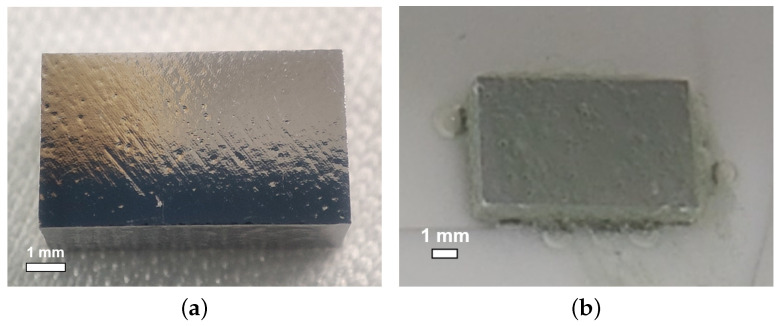
(**a**) The surface state of large-grain sample NL168 after polishing at an acid ratio of 3:1:6; (**b**) large-grain sample NL169 during polishing at an acid ratio of 3:1:9. The acid temperature is set at 8 °C.

Increasing the phosphoric acid ratio from 3:1:1 to 3:1:6, the reaction rate decreased as a power law to about 9.1 μm/min at 8 °C, as shown in Figure 5b. The polished surface changed from a scaly pit structure to a dotted pit structure containing dense small pits and sparse large circular pits, as shown in Figure 6a. At the low temperature of 8 °C and a ratio of 3:1:9, the samples shook slightly, and dense bubbles with different sizes and flocculent green products covered the surface of these samples, showing a slight boiling phenomenon on the surface, as shown in Figure 6b. Thus, a high phosphate ratio contributes to bubble formation and adhesion on the sample surface.

Although bubbles are formed with acid ratios ranging from 3:1:1 to 3:1:9, bubbles on the sample surface were observed only at high phosphoric acid ratios, which indicates that high acid viscosity may be a key factor affecting the formation of bubbles and W-shaped pits on the surface.

**Figure 7:**
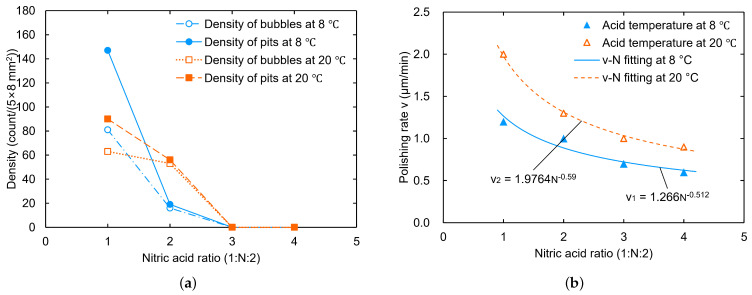
Effect of nitric acid ratio on bubble and pit density (**a**) and polishing rate (**b**) at acid ratio of 1:N:2.

Previous studies have shown that BCP with an acid ratio of 1:1:2 may cause the formation of W-shaped pits on the sample surface at both low temperature (8 °C) and room temperature (around 20 °C) [27]. Therefore, we tend to increase the nitric acid ratio to reach the goal of being bubbles- and pits-free. As shown in Figure 7, when increasing the nitric acid ratio from 1:1:2 to 1:4:2, the number of bubbles and W-shaped pits dramatically decreased until bubbles and pits were eliminated, in which the polishing rate obeyed a power law (as depicted before).

**Figure 8:**
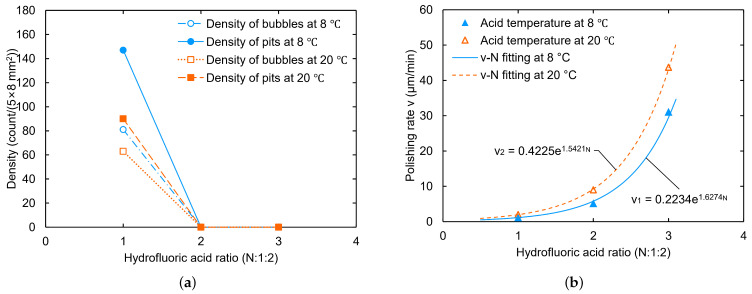
Effect of hydrofluoric acid ratio on bubble and pit density (**a**) and polishing rate (**b**) at acid ratio of N:1:2.

For the ratio N:1:2, a higher HF ratio also led to the absence of W-shaped pits, as shown in Figure 8. When the hydrofluoric acid ratio increased to 2:1:2 or higher, no bubble attachment or W-shaped pit formation was observed on the sample surface; however, it led to a boiling phenomenon, generating many bubbles. One can see that, although a high HF ratio may form bubbles very easily due to the high gas production rate, the lower acid viscosity and stronger surface disturbance will prevent these bubbles from attaching to the surface [34,37,41], thereby achieving a surface free of W-shaped pit formation.

#### 2.2.2. BCP Acid Ratios Without Pits

According to the above acid ratio experiments, the formation of bubbles and W-shaped pits is strongly related to the BCP acid ratio. In order to simply judge whether a BCP acid ratio leads to the formation of bubbles and W-shaped pits, we introduce a parameter, S, which is defined as the ratio of the volume sum of hydrofluoric and nitric acids to the volume of phosphoric acid, namely, S = V(HF+${\mathrm{HNO}}_{3}$)/V($H_{3}{\mathrm{PO}}_{4}$). The S value provides a reference for the optimization of the acid ratio, aiming to achieve pit-free polishing. Table 1 presents the S values under the experimental acid ratios.

According to the experimental results, when the parameter S ≥ 2 or the acid ratio (HF:${\mathrm{HNO}}_{3}$:$H_{3}{\mathrm{PO}}_{4}$) is 1:N:1, with acid temperature T ≤ 20 °C and the acid flow velocity at around 1.3 cm/s, no bubbles or W-shaped pits were observed on the niobium surface during BCP; however, if S ≤ 1, bubbles and pits were likely to occur, even at low temperature. In this case, the high acid viscosity and the strong suppression of disturbance or turbulence make it easy for bubbles to form and attach to the surface.

In adjusting or optimizing the BCP acid ratio, we suggest S ≥ 2 for safety. If the reaction rate needs to be adjusted, the ratio of the three acids can be increased or decreased on this basis; for example, 1:3:1, 1:4:1, or 1:3:2. Nevertheless, a large ratio of hydrofluoric acid and nitric acid is not recommended, as the surface quality after polishing may deteriorate due to excessive water content in the mixed acid [12,42]. For all of the above acid ratio experiments, the sample polishing rate versus acid ratio is also given.

In short, if the BCP acid ratio meets the condition S ≥ 2 or an acid ratio (HF:${\mathrm{HNO}}_{3}$:$H_{3}{\mathrm{PO}}_{4}$) of 1:N:1, through ensuring a low acid temperature (e.g., less than 20 °C) and appropriate acid flow velocity, a niobium surface free of W-shaped pits can be achieved.

### 2.3. A Superior BCP Acid Ratio

Aiming to achieve excellent RF performance, the BCP acid ratio for SRF cavity polishing not only requires no pit formation, but it must also be ensured that the polished niobium surface has no other obvious defects and the polishing process is controllable, with a moderate polishing rate.

In order to meet the requirements and achieve a polishing rate of 1–2 μm/min for cavity BCP-I, we selected a ratio of HF (49%):${\mathrm{HNO}}_{3}$ (69%):$H_{3}{\mathrm{PO}}_{4}$ (85%) at 1:3:1, yielding S = 4. The relatively higher nitric acid ratio can reduce the reaction rate and increase the dissolution rate of NO gas. In addition, excess nitric acid may be favorable for surface polishing, due to the formation of a thin layer of viscous niobium oxide [12,13].

**Figure 9:**
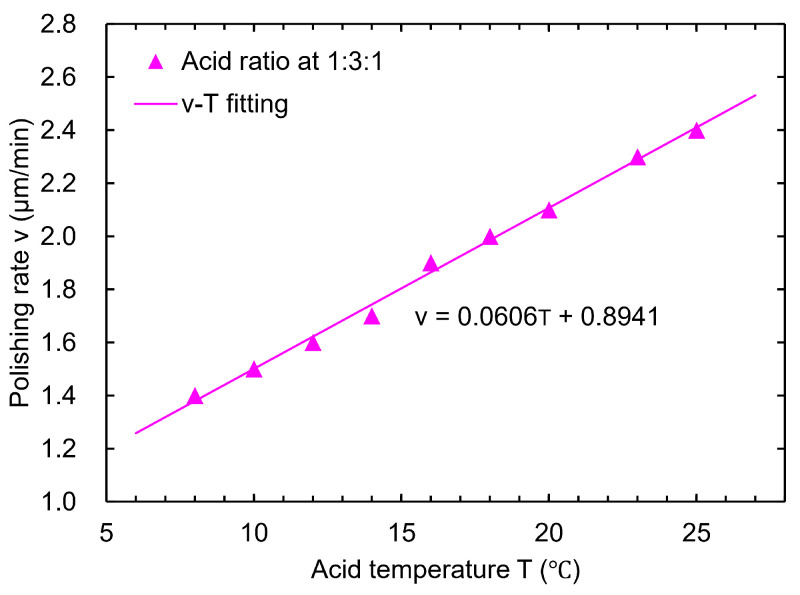
Polishing rates at different acid temperatures for the acid ratio 1:3:1.

**Figure 10:**
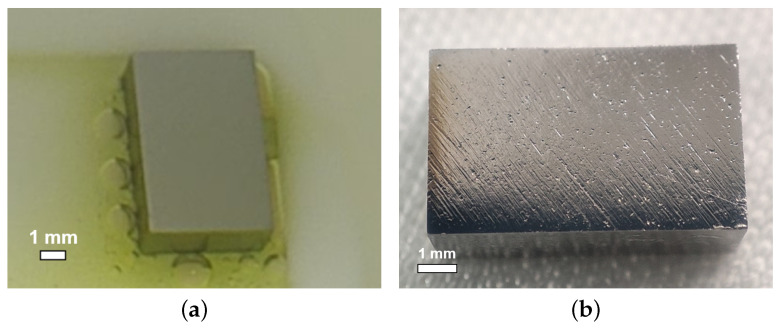
The surface states of large-grain sample NL175 during (**a**) and after (**b**) polishing, where the acid ratio is 1:3:1 and the acid temperature 25 °C.

As the applied polishing acid temperature varies in different laboratories, and as the acid temperature may rise rapidly after passing through the cavity (e.g., the acid outlet side of the cavity can reach higher than 15 °C when the inlet acid temperature is 8 °C), a new acid ratio guaranteeing no pit formation over a wide temperature range may be required. In our case, the surfaces of the samples were all free of bubbles and W-shaped pits at an acid ratio of 1:3:1 in the acid temperature range of 8–25 °C. The polishing rate shows a linear correlation with the acid temperature, reaching 1.4–2.0 μm/min in the range of 8–18 °C, as depicted in Figure 9. Figure 10 shows the polishing results for the ratio 1:3:1 at 25 °C, and the lack of bubble or W-shaped pit formation can be observed on the surface. Therefore, the new BCP acid ratio of 1:3:1 meets our requirements for sample BCP when the acid temperature is below 25 °C.

In order to verify the feasibility of the developed condition for real SRF cavities, we employed two single-cell niobium cavities (marked as L03 and S01) for BCP-I experiments, as shown in Figure 1b. The large-grain cavity L03 was subjected to light BCP for 10 min, after which it was determined that no W-shaped pits had formed in the cavity’s inner surface after polishing at the new acid ratio of 1:3:1, with an average polishing rate of about 1.4 μm/min as calculated according to the polishing removal mass. After that, the fine-grain cavity S01 was subjected to the BCP treatment with the new acid ratio of 1:3:1. The main treatment steps are as follows: (1) bulk BCP of 50 μm to reset the surface from previous treatments; (2) 900 °C heat treatment for 3 h in a vacuum furnace [43]; and (3) light BCP of 20 μm. The BCP process parameters for L03 and S01 were as follows: acid ratio of 1:3:1, acid temperature at around 8 °C, and acid flow rate of 10 L/min.

**Figure 11:**
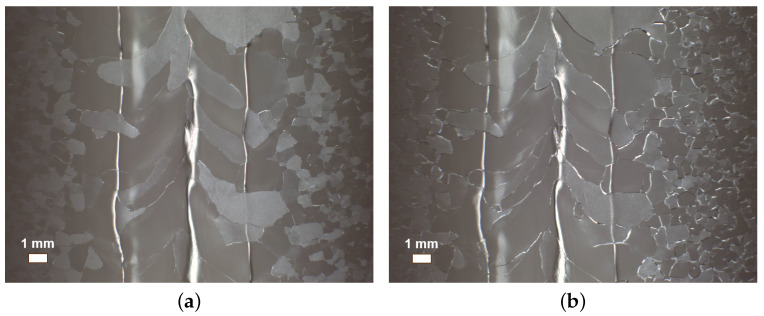
Optical inspection photographs of the equator region of the fine-grain cavity S01 (**a**) before and (**b**) after BCP (50 μm).

**Figure 12:**
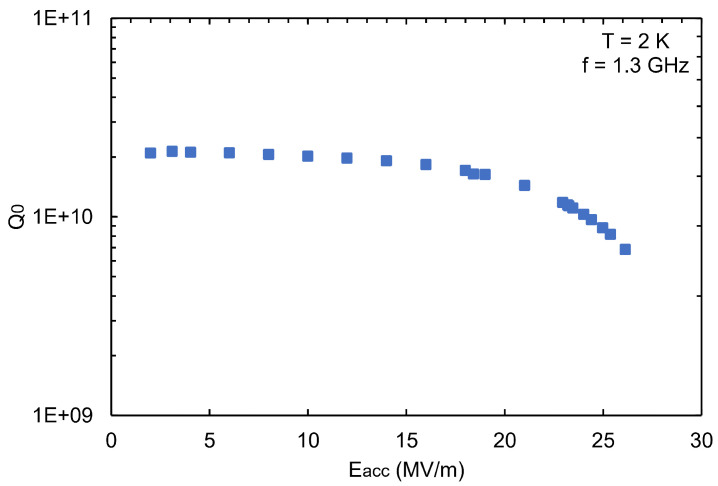
Vertical test results at 2 K of the fine-grain single-cell cavity S01 after standard BCP treatment with the new BCP acid ratio of 1:3:1.

Figure 11 shows the inner surface of cavity S01 before and after bulk BCP, where no W-shaped pit formation was observed, with a polishing rate of about 1.3 μm/min. The vertical test results are shown in Figure 12. The maximum accelerating gradient, $E_{max}$, of S01 reached 26.1 MV/m, with a reasonable intrinsic quality factor, demonstrating good RF performance.

As a result, we verified the successful BCP recipe with the new ratio of 1:3:1. This new BCP recipe brings pit-free formation, leading to a brilliant finish on the cavity surface, with a moderate polishing rate of about 1.3 μm/min. In the sample experiments, no bubbles or W-shaped pits appeared on the niobium surface, even when the acid temperature reached 25 °C. The ratio of 1:3:1 has been adopted as a standard BCP recipe at the Wuxi platform, and it has been applied for the polishing of several tens of SHINE 9-cell cavities, as well as components, accessories, and so on, since July of 2022.

## 3. Recipe Application

Table 2 details the optimized BCP-I processes for typical cavities at the Wuxi platform. A larger acid flow rate was used for the nine-cell cavities, in order to reduce the temperature difference between the bottom and the top of the cavity for better uniformity of polishing.

### 3.1. On 1.3 GHz Nine-Cell Cavity

**Figure 13:**
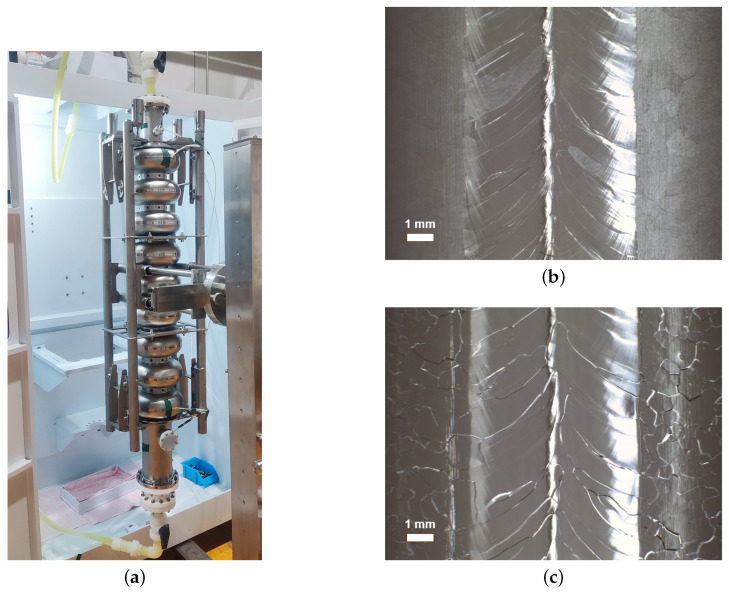
(**a**) BCP-I processing of twin-FPC cavity for SHINE project; (**b**) inner surface before and (**c**) after BCP (120 μm).

**Figure 14:**
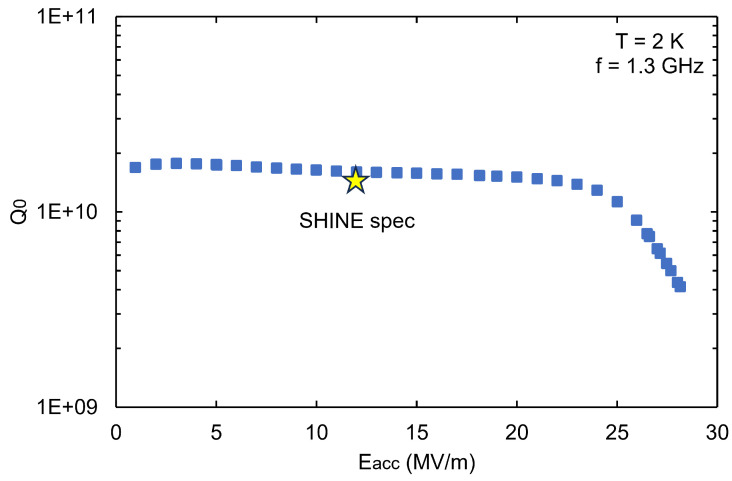
The vertical test results at 2 K of the twin FPC 1.3 GHz 9-cell cavity after standard BCP treatment with the new BCP acid ratio of 1:3:1.

A twin fundamental power coupler (FPC) cavity for the SHINE injector section was treated using the BCP recipe, which underwent bulk BCP of 120 μm, followed by 900 °C high-temperature baking in a furnace for 3 h, and finished with light BCP (20 μm), as shown in Figure 13a. Bulk BCP (120 μm) was performed to remove the surface damage layer caused during the machining and fabrication process [44,45]. High-vacuum heat treatment at 900 °C for 3 h was conducted to degas hydrogen and remove stress, and the light BCP (20 μm) removed the potentially contaminated layer formed during the heat treatment [2,8]. The inner surfaces before and after polishing are shown in Figure 13b,c. The average bulk and light polishing rates are about 1.3 and 1.5 μm/min, respectively. The higher polishing rate for light BCP was likely due to the fresh acid mixture, which contains less niobium. The vertical test result for this twin FPC cavity is shown in Figure 14, which reached $Q_{0}$ = 1.6 × $10^{10}$ at 12 MV/m and a maximum gradient of 28.2 MV/m, only limited by the insufficient input RF power.

### 3.2. On 3.9 GHz Nine-Cell Cavities

Two 3.9 GHz cryomodules with a total of 16 3.9 GHz nine-cell cavities are required for the SHINE accelerator to linearize the beam longitudinal phase space [46]. These 3.9 GHz nine-cell cavities were treated with the BCP recipe combined with two-step low-temperature baking [47], including BCP 130 μm + 900 °C/3 h + BCP 20 μm + 75 °C/4 h and 120 °C/48 h. The two-step baking process was employed to remove water from the surface and to further improve the accelerating gradient through modifying the surface oxide layer [2].

**Figure 15:**
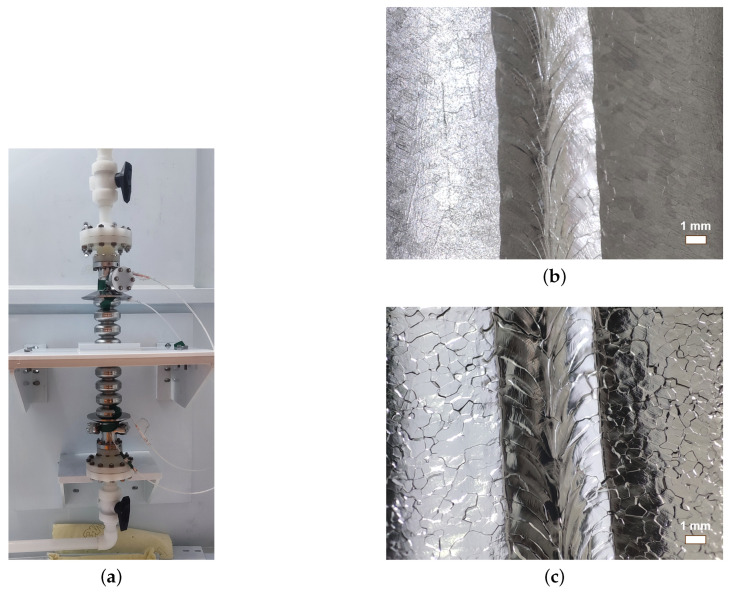
(**a**) BCP-I processing for the 3.9 GHz 9-cell cavity prototype H-NJ01; (**b**) the inner surface of H-NJ01 before and (**c**) after BCP (130 μm), captured by a self-made optical inspection machine [46].

**Figure 16:**
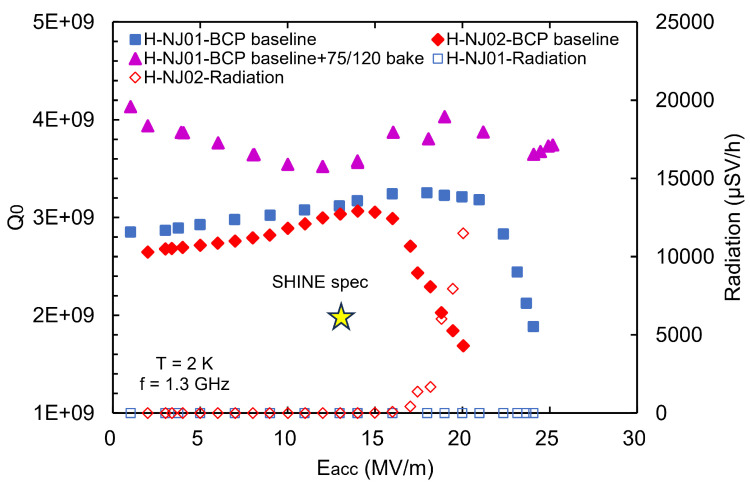
Vertical test result at 2 K of the two 3.9 GHz 9-cell cavity prototypes.

In order to verify the new BCP recipe for the 3.9 GHz cavities, two prototype nine-cell cavities were first treated with the BCP baseline without the two-step baking process, as shown in Figure 15a. The inner surface states before and after BCP-I polishing are shown in Figure 15b,c, respectively. The inner surface of H-NJ01 after polishing was flat and brilliant, as well as free of obvious defects. The average bulk polishing rate of the two cavities was about 1.4 μm/min. As shown in Figure 16, the $Q_{0}$ of the two cavities reached 3.0 ×$10^{9}$ at 13.1 MV/m, and the maximum gradient exceeded 20 MV/m; among them, H-NJ02 was limited by field emissions. The RF performances of these two prototypes are comparable to the performance of the 3.9 GHz cavities used in the EXFEL and LCLS-II projects [4,48]. Additionally, H-NJ01 was subjected to 75 °C/4 h + 120 °C/48 h baking. After the two-step baking process, the $Q_{0}$ of H-NJ01 was improved from 3.1 ×$10^{9}$ to 3.6 ×$10^{9}$ at 13.1 MV/m, and the maximum gradient increased from 24.1 MV/m to 25.2 MV/m, as shown in Figure 16.

Due to the excellent performance of the prototype cavities, the new BCP-I recipe was applied for the production of 3.9 GHz series cavities for the SHINE project. All 3.9 GHz nine-cell cavities were treated with the BCP baseline; among them, the first 15 cavities were subjected to two-step baking, while the other 4 cavities were treated without two-step baking due to our tight time schedule (H-NB06/07/08/09). The average bulk polishing rate of the 19 cavities was about 1.3 μm/min.

**Figure 17:**
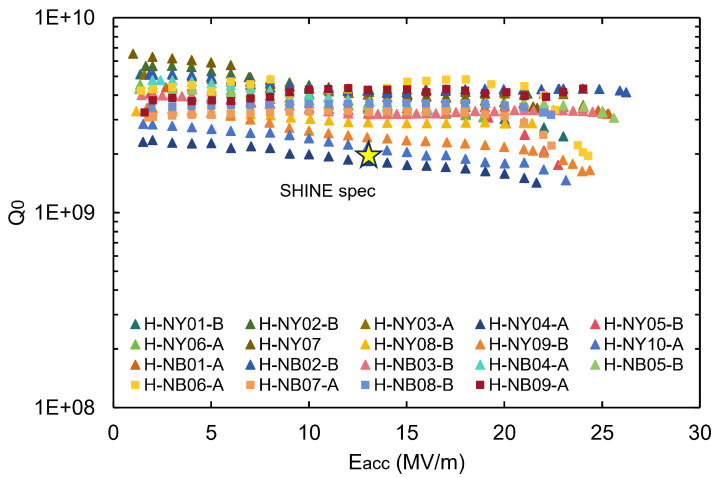
The vertical test performance at 2 K of the 19 3.9 GHz 9-cell cavities, including 18 jacketed cavities and 1 bare cavity (H-NY07). Suffixes A and B denote two versions of dressed cavities.

The vertical test results of these 19 cavities are shown in Figure 17; among them, H-NY07 is waiting for He-tank welding. The average $Q_{0}$ at 13.1 MV/m was 3.5 ×$10^{9}$, and the average maximum accelerating gradient was 23 MV/m, meeting the SHINE specifications. The $Q_{0}$ at maximum gradient for most of the 19 cavities was higher than 2 ×$10^{9}$, indicating the possibility to operate at a high gradient with acceptable cryogenic dissipation. Among them, four cavities (H-NB01/02/03/05) reached higher than 25 MV/m, demonstrating unprecedented RF performance for 3.9 GHz nine-cell cavities.

The two-step baking process is known as a high-gradient recipe that not only eliminates the high-field Q slope but also slightly enhances the $Q_{0}$. Although the performance of most cavities is a combination result of both BCP and two-step baking, the new BCP recipe provides a good baseline result, as can be seen from the results for the last four cavities. The high acceleration gradient of these cavities provides a large margin for SRF cavities in the module and the potential for mutual backup in the case of individual cavity failures.

## 4. Discussion

We addressed the problem of W-shaped pit formation during cavity BCP processing through changing the BCP acid ratio, thus providing a method and criteria for optimizing the BCP acid ratio, such that a superior BCP acid ratio can be obtained according to various demands. For different laboratories, the superior BCP acid ratio may differ, according their own conditions and demands (e.g., different concentrations of the three acids, different desired polishing rates, different surface quality requirement). The proposed ratio of 1:3:1 can be used as a reference. The method for changing the acid ratio is simple and widely applicable, and it does not require additional auxiliary devices or modifications to the BCP equipment.

During sample and cavity BCP processing, if the acid flow is too slow at some localized locations (e.g., the bottom of the sample, the fundamental power coupler port, the higher-order mode (HOM) coupler, and the pickup coupler ports of the Tesla cavity [2]), the produced NO gas may be trapped, which may result in bubbles and W-shaped pit formation even at an optimized BCP acid ratio. For instance, with some good acid ratios, even if no bubbles form on the sample surface during BCP polishing, large bubbles may still form around the sample, as shown in Figure 10a. In addition, air or NO gas accumulation at some local positions of a cavity—especially the low-β cavity with complex structure [2]—is highly likely to form pit imprints locally. Therefore, it is important to ensure a sufficient acid flow or exchange of acid on all cavity surfaces, such as adding branches for acid circulation at the fundamental power coupler, the HOM couplers, and pickup coupler ports for Tesla-shape cavity BCP-I.

Based on the formation mechanism of W-shaped pits, bubble formation is related to the gas production and dissolution rates, while bubble attachment is related to the viscosity and perturbation of the acid [27]. A low dissolution rate makes the produced gas unable to dissolve in time, while high acid viscosity hinders the diffusion of gas or movement of bubbles [31,34]; as such, gas aggregation and bubble attachment may ultimately result in the formation of pits on the surface.

The effect of the acid ratio 1:3:1 on the micro-morphology and composition of the niobium surface requires further material characterization. Whether there are other better acid ratios is also under investigation.

## 5. Conclusions

We systematically investigated the method involving changing the BCP acid ratio through sample and cavity BCP experiments, in order to solve the problem of W-shaped pit formation during cavity BCP processing. The results demonstrated that the ratio of the three acids has a significant role in the formation of bubbles and W-shaped pits on the niobium surface during BCP. We found that bubble and W-shaped pit formation can be avoided when the BCP acid (HF (49%):${\mathrm{HNO}}_{3}$ (69%):$H_{3}{\mathrm{PO}}_{4}$ (85%)) ratio meets the condition 1:N:1, where N is a positive number, or the volume ratio of the sum of HF and ${\mathrm{HNO}}_{3}$ to $H_{3}{\mathrm{PO}}_{4}$ is no less than 2, when the acid temperature is lower than room temperature and an appropriate acid flow velocity is used (around 1.3 cm/s). The proposed acid ratio at 1:3:1 was verified through single-cell cavities and was applied to both 1.3 GHz and 3.9 GHz nine-cell cavities for use in the SHINE project, with a polishing rate of around 1.3–1.5 μm/min. The resulting cavities were free of W-shaped pits and presented excellent RF performance.

## 6. Patents

CN2023114501762 was published on 12 January 2024 and is currently undergoing substantive examination.

## Figures and Tables

**Table 1:** S values for the experimental acid ratios and the corresponding bubbles observed on the sample surfaces. When the acid temperature T ≤ 20 °C, the acid flow velocity is about 1.3 cm/s.

Acid Ratio ^a^	Bubbles	S ^b^	Acid Ratio	Bubbles	S	Acid Ratio	Bubbles	S
3:1:3[1:$\frac{1}{3}$:1]	None	1.33	1:9:0	None	-	1:1:2	Yes	1
2:1:2[1:$\frac{1}{2}$:1]	None	1.50	1:4:0	None	-	1:1:$\frac{12}{5}$	Yes	0.83
1:1:1	None	2	3:1:1	None	4	3:1:6[1:$\frac{1}{3}$:2]	None	0.67
1:2:1	None	3	2:1:1	None	3	1:1:4	Yes	0.50
1:3:1	None	4	1:4:2	None	2.50	3:1:9	Yes	0.44
1:4:1	None	5	1:3:2	None	2	1:1:6	Yes	0.33
1:6:1	None	7	3:1:2	None	2	1:1:8	Yes	0.25
1:8:1	None	9	1:2:2[$\frac{1}{2}$:1:1]	Yes	1.50			
			1:3:3[$\frac{1}{3}$:1:1]	Yes	1.33			

^a^ Acid ratio HF (49%):${\mathrm{HNO}}_{3}$ (69%):$H_{3}{\mathrm{PO}}_{4}$ (85%). ^b^ S = Volume (HF+${\mathrm{HNO}}_{3}$)/Volume ($H_{3}{\mathrm{PO}}_{4}$).

**Table 2:** Parameters of standard BCP-I processes for cavities used at Wuxi platform after July of 2022.

Cavity Types	Acid Ratio ^a^	Acid Temperature (°C)	Acid Flow Rate (L/min)	Bulk Polishing Rate (μm/min)
1.3 GHz one-cell	1:3:1	8	10	1.3
1.3 GHz nine-cell	1:3:1	8	20	1.3
3.9 GHz one-cell	1:3:1	8	1.5	-
3.9 GHz nine-cell	1:3:1	8	2.5	1.3

^a^ Acid ratio HF (49%):${\mathrm{HNO}}_{3}$ (69%):$H_{3}{\mathrm{PO}}_{4}$ (85%).

## Data Availability

The original contributions presented in this study are included in the article. Further inquiries can be directed to the corresponding authors.

## Abbreviations

The following abbreviations are used in this manuscript:

BCP	buffered chemical polishing
BCP-O	outer-surface BCP
BCP-I	inner-surface BCP
SRF	superconducting radio-frequency
RRR	Residual Resistivity Ratio
HPR	High-Pressure Rinsing
RPM	Revolutions Per Minute
FPC	fundamental power coupler
HOM	higher-order mode
